# An Unusual Phage Repressor Encoded by Mycobacteriophage BPs

**DOI:** 10.1371/journal.pone.0137187

**Published:** 2015-09-02

**Authors:** Valerie M. Villanueva, Lauren M. Oldfield, Graham F. Hatfull

**Affiliations:** Department of Biological Sciences, University of Pittsburgh, Pittsburgh, Pennsylvania, 15260, United States of America; Niels Bohr Institute, DENMARK

## Abstract

Temperate bacteriophages express transcription repressors that maintain lysogeny by down-regulating lytic promoters and confer superinfection immunity. Repressor regulation is critical to the outcome of infection—lysogenic or lytic growth—as well as prophage induction into lytic replication. Mycobacteriophage BPs and its relatives use an unusual integration-dependent immunity system in which the phage attachment site (*attP*) is located within the repressor gene (*33*) such that site-specific integration leads to synthesis of a prophage-encoded product (gp33^103^) that is 33 residues shorter at its C-terminus than the virally-encoded protein (gp33^136^). However, the shorter form of the repressor (gp33^103^) is stable and active in repression of the early lytic promoter P_R_, whereas the longer virally-encoded form (gp33^136^) is inactive due to targeted degradation *via* a C-terminal ssrA-like tag. We show here that both forms of the repressor bind similarly to the *33*–*34* intergenic regulatory region, and that BPs gp33^103^ is a tetramer in solution. The BPs gp33^103^ repressor binds to five regulatory regions spanning the BPs genome, and regulates four promoters including the early lytic promoter, P_R_. BPs gp33^103^ has a complex pattern of DNA recognition in which a full operator binding site contains two half sites separated by a variable spacer, and BPs gp33^103^ induces a DNA bend at the full operator site but not a half site. The operator site structure is unusual in that one half site corresponds to a 12 bp palindrome identified previously, but the other half site is a highly variable variant of the palindrome.

## Introduction

Following adsorption and DNA injection, temperate phages must choose between two alternative outcomes: lytic growth in which the phage replicates and the cell lyses to release progeny phage particles, or lysogeny in which the lytic genes are switched off and a prophage genome is maintained either by site-specific chromosomal integration, or stable extrachromosomal replication [1]. In the well-studied example of phage lambda, lysogenic maintenance is achieved by expression of a repressor (cI) that binds to tripartite operators (O_L_ and O_R_) at the early lytic promoters P_L_ and P_R_ [1]. Lambda cI autoregulates its synthesis by activation of its own transcription from the promoter for lysogenic maintenance (P_RM_) at moderate cI concentrations, and represses it when the cI concentration is high. During infection, establishment of lambda lysogeny occurs by expression of cI from the promoter for lysogenic establishment (P_RE_), which is independent of cI, but requires the activator, cII [2]. The decision as to the outcome of infection is determined by the overall level of cII, which is subject to degradation by host proteases including FtsH, and is modulated by lambda cIII protein [3]. Lambda cI binds as a dimer and can form DNA loops when bound at both O_L_ and O_R_ [4].

The temperate life style is common among bacteriophages, although the genetic diversity of the phage population is considerable [5]. Repressors have been identified in many phage genomes, although relatively few have been genetically and biochemically characterized [5]. The organization of two divergently transcribed DNA-binding proteins—typified by the *cI* and *cro* genes in lambda—separated by a control region is common but not universal. For example, the repressor of *Streptomyces* phage ϕC31 is located downstream of the virion structural genes and is similarly transcribed rightwards [6, 7], and in mycobacteriophage L5 (and its relatives) the repressor is located within the right arm of the genome and transcribed leftwards along with other right arm genes [8, 9]. These two systems are also unusual in that the phage genomes contain multiple (18–30) repressor binding sites dispersed across the genomes, and the ϕC31 system is further complicated by the expression of three isoforms of the repressor [10]. The L5 repressor binds as a monomer at the asymmetric non-operator binding sites (referred to as ‘stoperators’) to block transcription elongation [11]. There are few examples other than phage lambda and its relatives where the molecular basis of the decision between lytic and lysogenic outcomes is well understood [12, 13].

Comparative genomic analysis of a large number of completely sequenced mycobacteriophages shows them to be highly diverse, and they can be grouped into ‘clusters’ according to their nucleotide and gene content relationships [14, 15]. A substantial portion of these phages (~40%), including L5, are grouped in Cluster A, and share the unusual stoperator system of immunity [11, 16]. The repressor genes have been identified in phages of Clusters G, I, N, and P [12, 13], Cluster K [17], and the singleton Giles [18], but in each case they are components of pairs of divergently transcribed genes separated by a putative control region.

**Fig 1:**
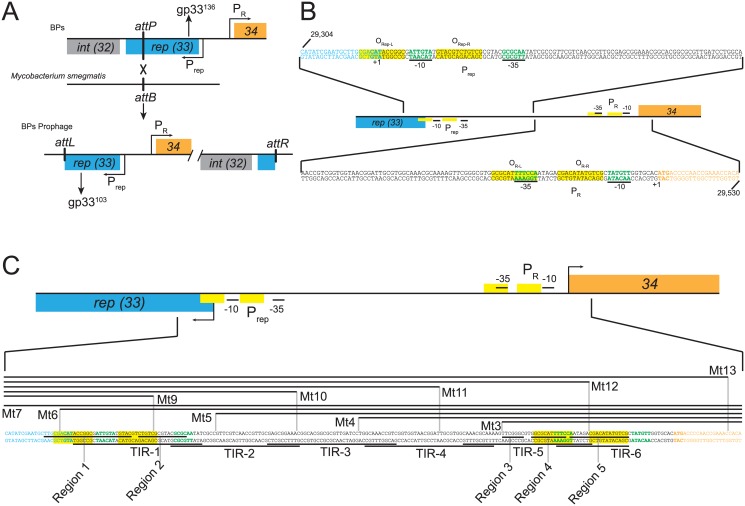
The BPs Immunity Cassette. (A) Phage BPs has three genes involved in the lytic/lysogenic growth decision; *gene 32* encodes an integrase (gp32), *gene 33* encodes the repressor (gp33), and *gene 34* encodes a putative Cro-like protein (gp34). BPs gp33 and gp32 are expressed leftwards from the P_rep_ promoter and gp34 is expressed rightward from the P_R_ promoter. The *attP* site for BPs integration is within the *33* open reading frame, such that the prophage repressor gene is truncated the gene by 99bp. The virally-encoded repressor is 136 residues long (gp33^136^) and has a C-terminal tag for proteolytic degradation [13]. This prophage form of the repressor (gp33^103^) is 33 residues shorter, is stably expressed, and is required to maintain lysogeny. (B) The *33–34* intergenic region contains two divergent promoters, P_rep_ and P_R_. The full intergenic region sequence is shown (left part above, right part below) and promoter -10 and -35 elements are in green. The operator (O_R_; highlighted in yellow) to which gp33^103^ binds to regulate P_R_ is located between the -10 and -35 elements of P_R_ (green). A second operator, O_Rep_, is located proximal to *33* (highlighted in yellow) downstream of the P_rep_ promoter (green). (C) DNA included in substrates used for binding assays are shown as horizontal lines above or below the *33*–*34* intergenic sequence (Mt series and TIR series of substrates). Regions of DNase I protection are indicated by black lines between the top and bottom strand bases.

Mycobacteriophage BPs and its relatives in Cluster G—along with members of Clusters I, N, and P—use an unusual integration-dependent immunity system for the establishment and maintenance of lysogeny [13]. In these systems the repressor and integrase genes (BPs *32* and *33* respectively) are transcribed leftwards, but the phage attachment site (*attP*) is oddly located within the repressor open reading frame (see Fig 1A). As a consequence, chromosomal integration in lysogenic establishment results in separation of the 3’ end of the repressor gene and expression of a truncated version of the repressor. However, it is this short form of the repressor (e.g. BPs gp33^103^) that is active in conferring immunity, whereas the virally-encoded longer form (e.g. BPs gp33^136^) is not. Inactivation of the virally-encoded form expressed during lytic growth occurs as a result of proteolytic degradation targeted at an ssrA-like tag at the extreme C-terminus [13]. Proteolysis of the virally-encoded form is a direct determinant of lysogenization frequency, as a mutant expressing a stabilized form of BPs gp33^136^ establishes lysogeny at a considerably higher frequency than wild-type BPs. However, lysogenization frequency is also determined by the frequency of integration, and the integrase protein also contains a C-terminal signal for proteolysis [13].

The BPs *33*–*34* intergenic region contains two divergent promoters, P_R_ and P_rep_, responsible for early lytic expression and repressor synthesis respectively [13, 19] (Fig 1B). The gp33^103^ active form of the repressor was shown previously to bind to a DNA substrate that includes a 12 bp palindromic sequence, which was presumed to be the operator (O_R_) regulating transcription from the early lytic promoter, P_R_ [13]. This was supported by the mapping of two point mutations within this 12 bp sequence (5’-CGACATATGTCG) that give rise to a repressor-insensitive phage phenotype (i.e. they can form plaques on a repressor-expressing strain) [13]. However, the requirements for DNA binding at O_R_ and at related sequences elsewhere in the phage genome and the nature of the protein-DNA interactions are not understood. Here we show that the two forms of the repressor bind similarly to DNA, that the short active form of the repressor is a tetramer in solution, and that the previously reported 12 bp operator sequence represents one half of a full binding site.

## Results

### gp33^136^ and gp33^103^ bind similarly to BPs *33–34* intergenic DNA

**Fig 2:**
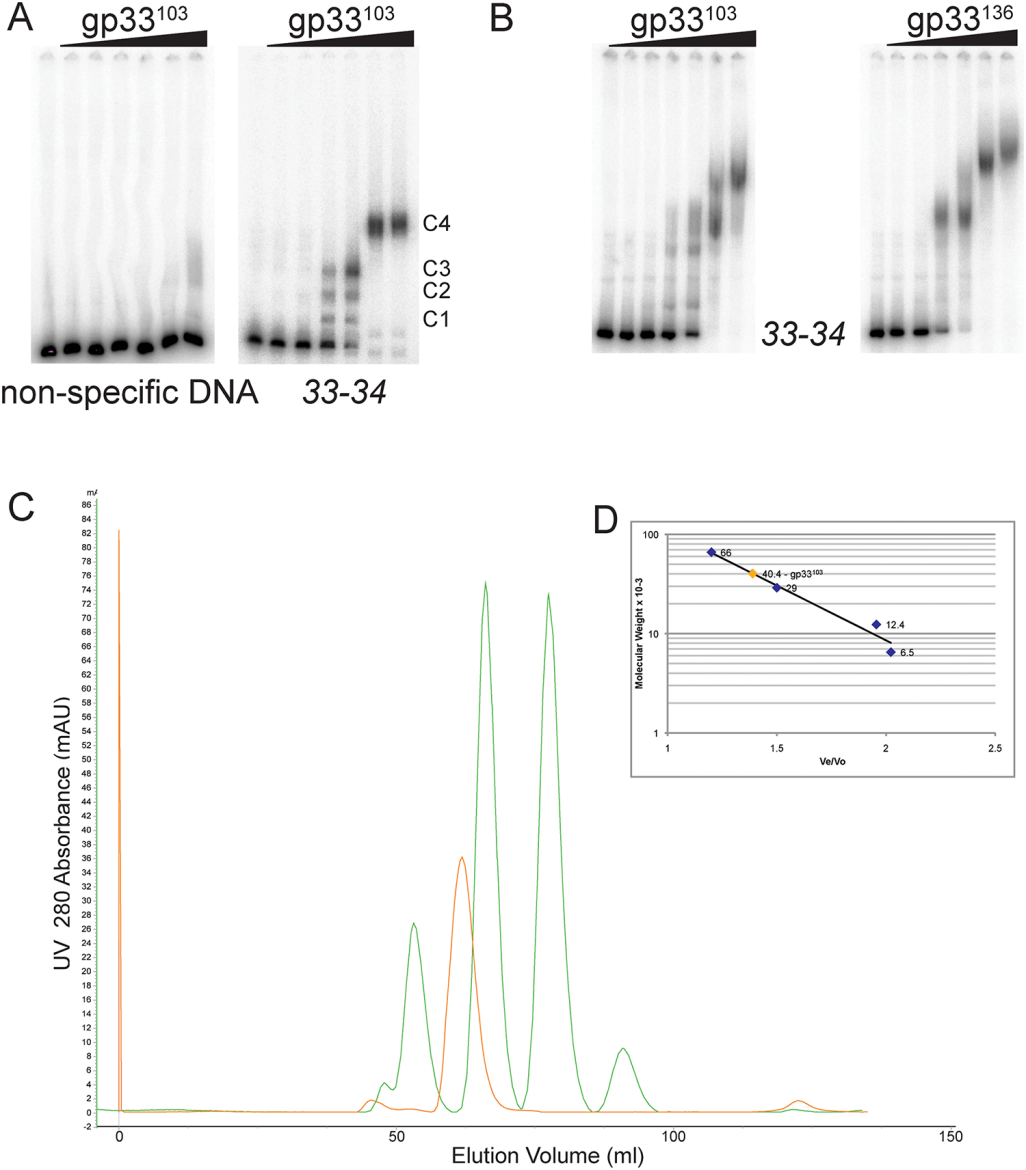
Both forms of gp33 bind similarly to the BPs *33–34* intergenic region. (A) EMSA analysis shows that gp33^103^ binds to the *33*–*34* intergenic region with a complicated pattern of binding with four distinct complexes, labeled C1, C2, C3, and C4. No binding was seen between gp33^103^ and a non-speciic DNA substrate. Protein concentrations are as follows: 1) none 2) 0.16 μM 3) 0.54 μM, 4) 1.6 μM, 5) 5.4 μM, 6) 16 μM, 7) 54 μM. (B) DNA binding assays show that both gp33^103^ and gp33^136^ bind similarly to a DNA substrate containing PCR amplified DNA from the BPs gene *33–34* intergenic region (S1 Table). Protein concentrations are as shown in A. (C) Size-exclusion chromatograms for gp33^103^ (orange) and the molecular weight markers (green; 66kDa, 29kDa, 12.4kDa, 6.5kDa) are overlayed. (D) The inset graph shows a standard plot for the molecular mass for the protein standards against the ration of their elution volume (Ve) and void volume (Vo) (blue diamonds); the molecular mass of gp33^103^ is predicted to be 40.4kDa (orange diamond).

Electrophoretic mobility shift assays (EMSA) show that gp33^103^ binds to DNA substrates containing the *33*–*34* intergenic control region to form several distinct complexes as protein concentration increases (Fig 2A). The combined affinity for gp33^103^ binding is relatively weak (2.6 μM; Table 1) and the prominent complex (C4) forms at protein concentrations of 16 μM and above (Fig 2A); three faster migrating complexes are observed at lower protein concentrations and are likely binding intermediates (Fig 2A). Although DNA binding is relatively weak compared to other repressors, it is specific as little or no binding is observed with a control substrate (Fig 2A), and the binding reactions all contain 1 μg calf thymus DNA.

**Table 1:** Binding affinities of gp33^103^ to various DNA substrates.

Mutant Name	Mutation in *33–34* region	Average K_d_ (Molar)
Wild type *33–34* region	None	2.61 x 10^−6^
102a	T29489C	7.05 x 10^−6^
102e	A29486C	7.80 x 10^−6^
102j	151bp Inversion (29282–29432)	4.23 x 10^−6^
102k	24bp Duplication (29472–29495)	6.67 x 10^−6^
127b	151bp Deletion (29323–29473)	9.50 x 10^−6^
127c	33bp Duplication (29495–29527)	3.50 x 10^−6^
127d	12bp Duplication (29474–29485)	2.99 x 10^−6^
127e	6bp Duplication (29483–29488)	3.81 x 10^−6^
Δ32 Clr1	T29336C	1.74 x 10^−6^
Δ32 Clr4	G29500A	1.45 x 10^−6^
33A129E Clr5	C29372T	2.90 x 10^−6^
33A129E Clr6	T29475C	2.65 x 10^−6^
Δ32 Clr8	T29501C	2.80 x 10^−6^
*5–6* intergenic region	None	2.75 x 10^−6^
*26–27* intergenic region	None	9.00 x 10^−7^
*54–55* intergenic region	None	2.60 x 10^−6^
*60–61* intergenic region	None	3.40 x 10^−6^
O_6-L_	None	1.05 x 10^−5^
O_27-R_	None	1.10 x 10^−5^
O_27-L_	None	3.85 x 10^−5^
O_55-L_	None	8.50 x 10^−6^
O_61-R_	None	1.95 x 10^−5^
TIR-5	None	1.25 x 10^−5^
TIR-6	None	9.50 x 10^−6^

BPs gp33^136^ is longer than gp33^103^ because of an additional 33 residues at its C-terminus. The C-terminal extension includes the ssrA tag that targets the protein for proteolysis, and stabilization of the protein by an A135E substitution gives higher levels of lysogeny. However, we noted previously that the gp33^136^ A135E mutant appears to give a modest increase in activity of the P_rep_ promoter in a reporter fusion assay [13], perhaps providing transcriptional activation that is dependent on the C-terminal 33 residues. We therefore compared the binding profiles of gp33^103^ and gp33^136^ for any differences in binding to the *33*–*34* intergenic region that contains both P_rep_ and P_R_ (Figs 1 and 2B). Both proteins give similar profiles and the additional 33 C-terminal residues in gp33^136^ do not appear to substantially influence DNA binding. It is likely that any functional activation of P_rep_ results from protein-protein interactions, perhaps reflecting direct contacts between BPs gp33^136^ and RNA Polymerase.

The 185 bp DNA segment contains only a single copy of the 12 bp sequence 5’-CGACATATGTCG that was previously predicted to be recognized by gp33^103^, and yet the complexes formed are more varied than would be expected for a single protein-DNA interaction. Presumably, the protein binds to additional sites within this region, binds with varying protein-DNA stoichiometries, or imposes significant DNA distortions. We note that there is a related sequence (5’-CGACATACCGGC) at the left end (*33*-proximal) of the intergenic region that overlaps the P_rep_ transcription start site (Fig 1B), although the similarity is restricted to the left half of the sequence motif. We therefore sought to dissect the various determinants of gp33^103^ binding to this region, and to compare this to the binding of gp33^103^ to the other sites located elsewhere in the BPs genome.

### Solution multimeric state of BPs gp33^103^

First, we determined the oligomeric state of gp33^103^ in solution. A single protein peak was observed using size-exclusion chromatography, and when compared with protein markers, has an apparent molecular mass of 40.4 kDa (Fig 2C). The monomeric mass of gp33^103^ is 11.2 kDa, and the simple interpretation is that the major peak corresponds to a gp33^103^ tetramer, although we cannot rule out the possibility that it is an alternative multimer shaped to give altered elution in the chromatography. We note that the 12 bp operator described previously [13] has dyad symmetry (Fig 1B) consistent with recognition by either a dimer or tetramer of gp33^103^. The gp33^103^ elution profile did not show any other prevalent forms and the oligomeric state is quite homogenous.

### DNase I footprinting of the *33–34* intergenic region

**Fig 3:**
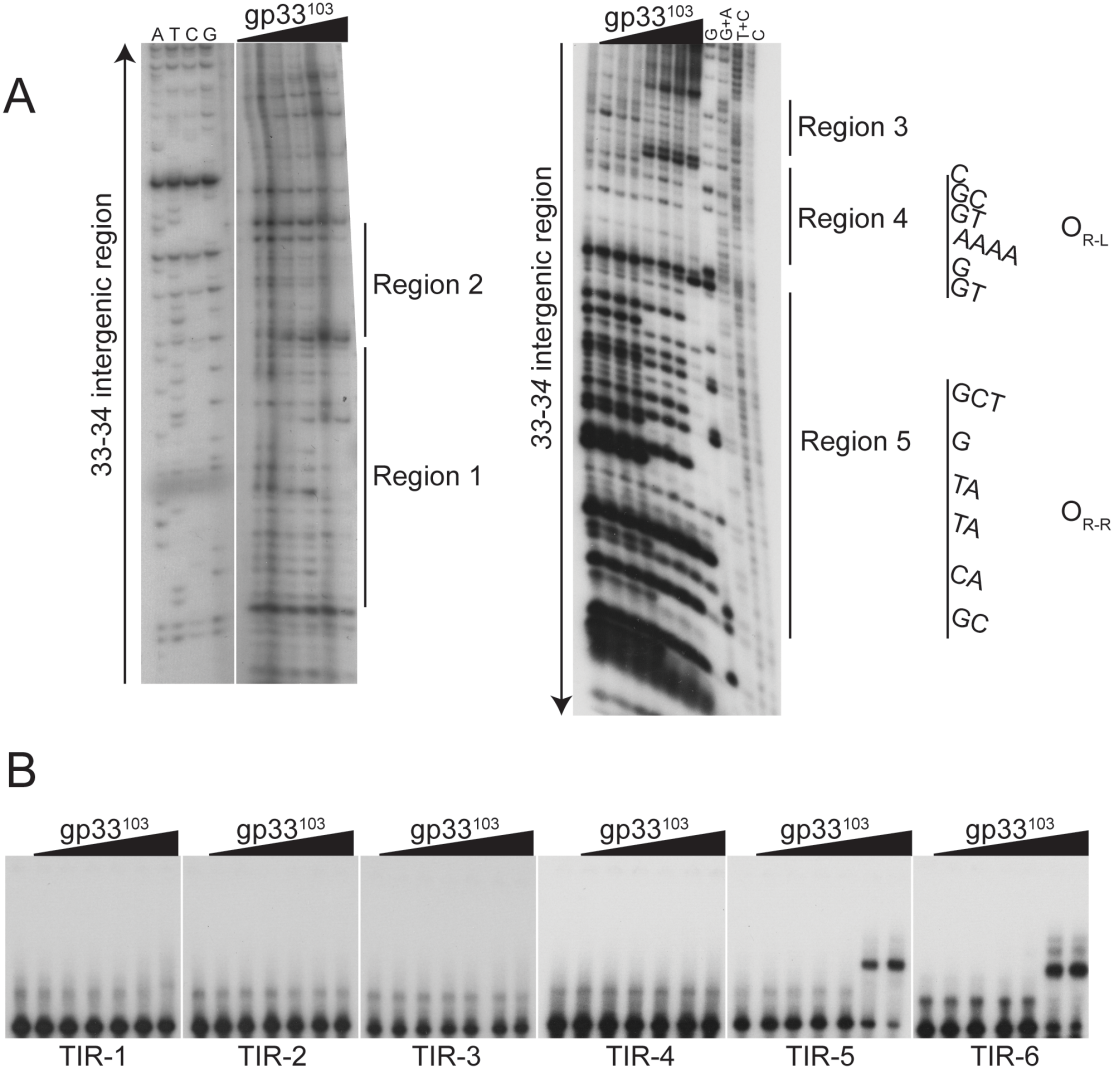
BPs gp33^103^ binds to several regions within the *33–34* intergenic region. (A) DNase I footprinting assays (panels A and B) were done using 5’ end-labeled probes of the *33–34* intergenic region, which were incubated with increasing concentrations of gp33^103^, subjected to DNase I cleavage, and run on a denaturing gel alongside a sequencing ladder. Black arrows show the direction of the sequence from 5’ to 3’ for each DNA strand. Regions of protection from DNase I cleavage are indicated by black bars and labeled; the sequence of O_R_ is also shown. Protein concentrations are as follows: 1) none 2) 0.16 μM 3) 0.54 μM, 4) 1.6 μM, 5) 5.4 μM, 6) 16 μM, 7) 54 μM, 8) 160 μM. Two regions of protection observed near P_rep_, one of which contains O_Rep_ (Regions 1 and 2). Three regions of protection are observed near P_R_ (Regions 3 and 4, Region 5), one of which includes O_R-R_ (Region 5). The sequences of the protected regions are shown in Fig 1C. See also S1 Fig (B) EMSAs showing gp33^103^ binding to 40 bp substrates spanning the *33–34* intergenic region (see Fig 1C). Binding occurs to the TIR-6 fragment containing O_R-R_ and to substrate TIR-5, which contains Region 3 and Region 4 (O_R-L_) and only one base pair of O_R-R_. (A). Protein concentrations are as follows: 1) none 2) 0.16 μM 3) 0.54 μM, 4) 1.6 μM, 5) 5.4 μM, 6) 16 μM, 7) 54 μM.

DNase I footprinting provides further insights into the binding of gp33^103^ to the *33*–*34* intergenic region (Fig 3A). Depending on the gp33^103^ concentration, two different patterns of altered DNase I sensitivity are observed. At intermediate concentrations (5.4 μM– 54 μM) we see prominent protection from DNase I cutting at positions in and around the 12 bp palindromic sequence although some cut sites remain sensitive to DNase I cleavage (Fig 3A). However, there are notable enhancements of DNase I cutting to the left of the 12 bp palindrome situated approximately 20 bp and 30 bp away respectively (Fig 3A, S1 Fig). At the highest concentrations of protein used (160 μM), DNase I protection is more extensive within this region and extends to two regions (designated regions 3 and 4; Figs 1C and 3A) flanking the DNase I enhancement located about 20 bp to the left of the 12 bp palindrome. There is also protection in the gene *33* proximal end of the substrate in regions designated 1 and 2, with apparent DNase I enhancement between them (Figs 1C and 3A). As the conditions for DNase I footprinting and native gel electrophoresis of complexes are somewhat different it is not possible to draw a direct comparison between the two, although it is likely that the slowest moving of the protein-DNA complexes corresponds to the more extensive DNase I protections seen at the highest protein concentration, and that the faster migrating complexes correspond to those seen at intermediate concentrations (Figs 2A and 3A).

Because of these DNase I protection patterns together with the experiments described below, we propose that the gp33^103^ binding site at P_R_ spans a larger region than just the 12 bp palindrome, and that this palindrome is equivalent to a half site, of which region 4 (and perhaps part of region 3) constitute the other half site, not withstanding the sequence dissimilarities (see Fig 1C). To simplify the presentation and discussion of data below, we will refer to the 12 bp palindrome as O_R-R_ and the region to the left that includes region 4 as O_R-L_ (Fig 1B) reflecting the right and left half sites of O_R_ respectively. The regions 1 and 2 of protection at P_rep_ will be referred to as O_Rep_.

### Binding of gp33^103^ to subsites in the *33–34* intergenic region

The binding of gp33^103^ at O_Rep_ seen by DNase I footprinting was somewhat unexpected as this region lacks a site equivalent to O_R_ or an obvious O_R_ half site (O_R-L_ or O_R-R_), although it has a distantly related sequence (Fig 1C). However, occupancy of the O_Rep_ site is evident at the highest protein concentration in DNase I footprinting (Fig 3) and may depend on concomitant binding of gp33^103^ elsewhere in the DNA fragment, such as at O_R_.

To further explore these interactions we synthesized a series of small (40 bp) dsDNA substrates, containing segments of the *33*–*34* intergenic region (Fig 1C) and asked whether gp33^103^ binds and forms protein-DNA complexes (Fig 3C). The protein binds to the TIR-6 substrate (Figs 1C and 3C) containing O_R-R_ to form a single complex, but binds similarly to the TIR-5 substrate to form complexes with similar mobilities (Fig 3C), which was unexpected as the TIR-5 substrate lacks the 12 bp palindromic sequence previously identified as O_R_ [13]. The TIR-5 substrate contains O_R-L_ but does not contain O_R-R_ (see Fig 1C). A plausible explanation is that gp33^103^ binds as a dimer or tetramer to O_R-L_ and O_R-R_ independently and at reduced affinity, notwithstanding the sequence differences. In this model, binding of a tetramer would involve two unoccupied helix-turn-helix DNA binding domains, which would be available for binding either to another site within the same substrate (perhaps O_Rep_) with introduction of a DNA loop, or by forming intramolecular bridges between two different DNA molecules.

### Binding of gp33^103^ to deletion substrates of the *33–34* intergenic region

**Fig 4:**
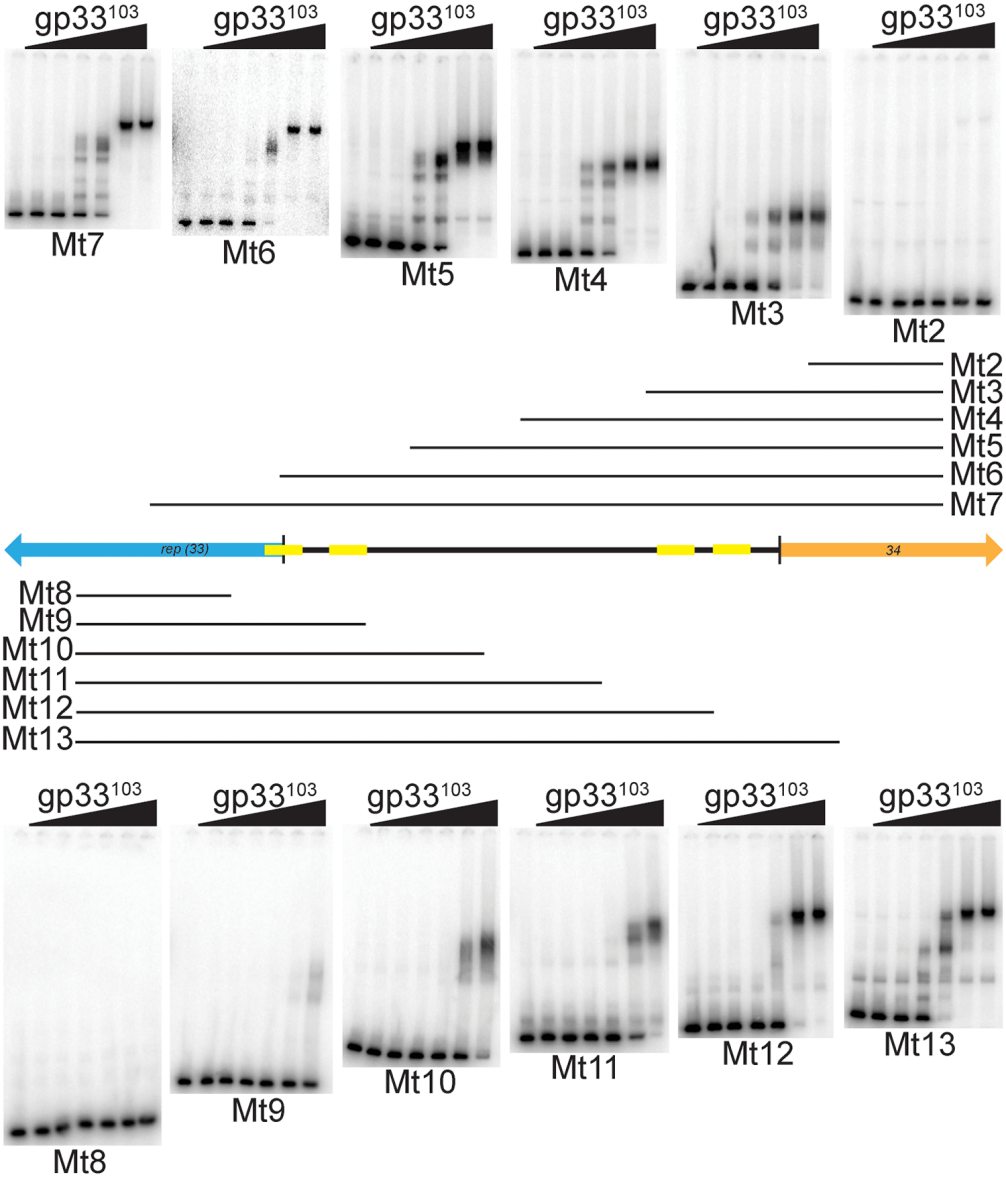
BPs gp33^103^ binds with variable affinities to a series of end-deletion substrates. Substrates were PCR amplified from the BPs *33–34* intergenic region using primers located at regular intervals from each end to sequentially shorten the *33–34* intergenic region from each end (S1 Table). The sequences included in each substrate are shown as horizontal black lines either above or below a schematic representation of the *33*–*34* intergenic region. Genes 33 and 34 are represented by blue and orange arrows respectively, and O_R_ and O_Rep_ are shown in yellow. Protein concentrations are same as shown in Fig 2A. Protein affinities are shown in Table 1.

To further examine the parts of the *33–34* intergenic region required for binding we generated a series of substrates containing progressive deletions from each end and tested them for binding of gp33^103^ (Fig 4). One notable observation is that the binding patterns with the Mt12 and Mt13 substrates (Figs 1C and 4) are similar, even though O_R-R_ is present in Mt13 but absent from Mt12. The affinity for Mt12 is slightly reduced relative to that for Mt13, but we assume that the slowest migrating complexes (C4) have similar stoichiometries, with each containing a dimer or tetramer of gp33^103^ bound at O_R_ in addition to binding elsewhere in the substrate. One of the Mt13 complexes (C3) appears to be absent from the Mt12 substrate, and presumably requires O_R-R_ (Figs 1 and 4). Complete removal of both O_R-L_ and O_R-R_ (substrates Mt10 and Mt11) does not completely eliminate binding and complexes are observed albeit with reduced binding affinity (Fig 4; Table 1), reflecting weaker binding to the left part of the substrate.

Deletion substrates lacking P_rep_-proximal regions but retaining O_R_ (i.e. Mt6 and Mt7) form similar complexes, although the major complexes have somewhat different relative mobilities (0.69, 0.55, and 0.50 for Mt3, Mt4, and Mt5 respectively), perhaps reflecting DNA distortions (Fig 4). Inclusion of either part (i.e. Mt6) or all (i.e. Mt7) of the putative O_Rep_ site imposes little overall change to the pattern of complex formation (Fig 4), even though DNase I footprinting (Fig 3A) shows that gp33^103^ binds at the higher protein concentration to substrate similar to Mt7; furthermore, gp33^103^ forms complexes with substrates Mt10 and Mt 11 that lack O_R_ (Fig 4). It is unclear whether gp33^103^ binds separately at O_R_ and O_Rep_ or if one or more tetramers of gp33^103^ bind simultaneously at O_Rep_ and O_R_ to form a DNA loop.

### BPs gp33^103^ introduces a DNA bend when bound to the *33–34* intergenic region.

**Fig 5:**
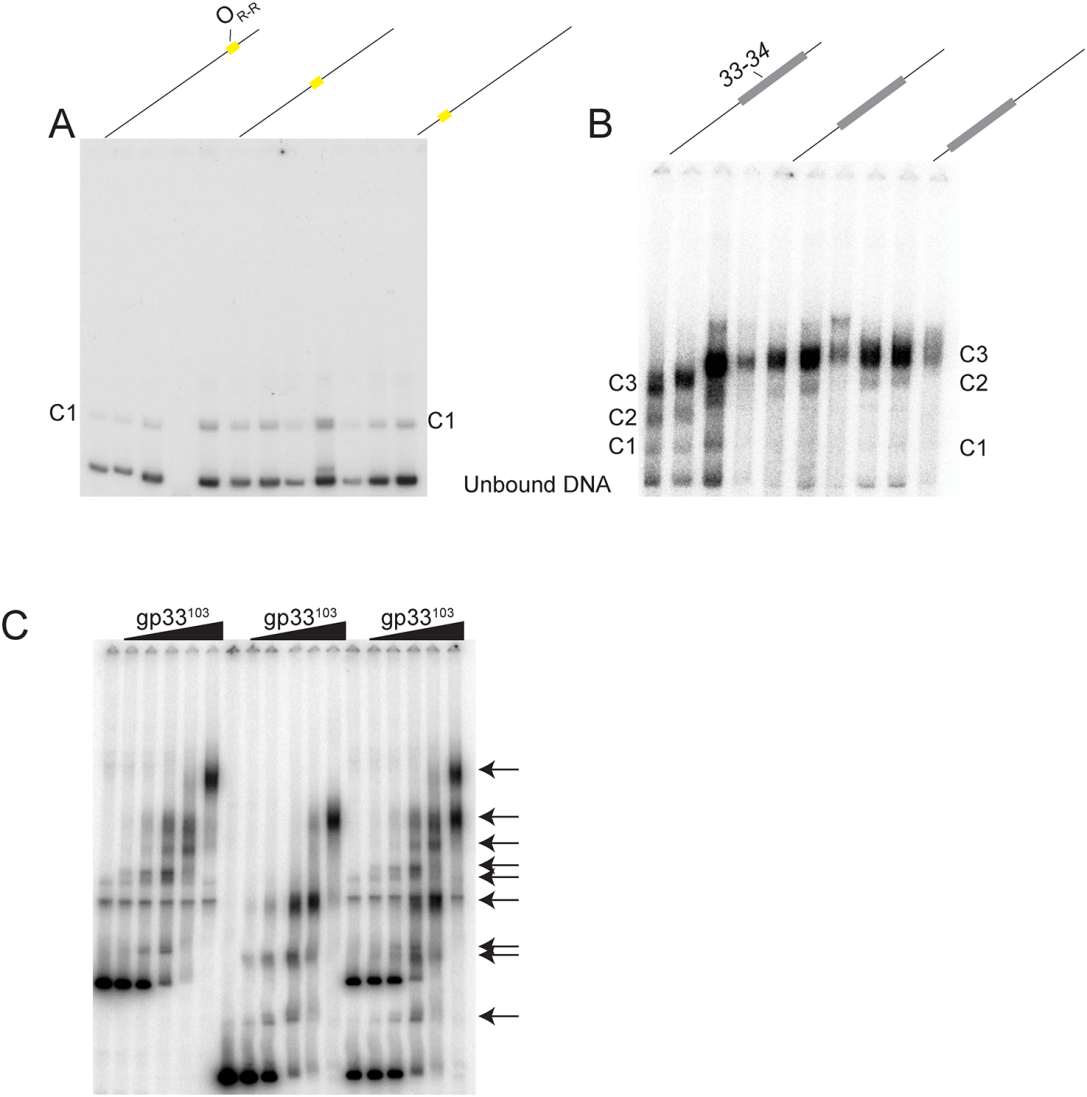
BPs gp33^103^ bends DNA but does not facilitate loop formation. (A) The 12bp of O_R_ was cloned into plasmid pBend2 [20] using unique restriction enzyme sites SalI and XbaI to create pVMV22. Equal-sized fragments of DNA were excised from pVMV22 with O_R_ located at different positions relative to the edge of the probe by cleaving with appropriate enzymes. BPs gp33^103^ (0.54μM) was incubated with each radiolabeled probe and run on a native polyacrylamide gel. The only gp33^103^-DNA complex (C1) does not show any differences in relative mobility with different substrates. (B) A second plasmid containing the entire BPs *33–34* intergenic region (pVMV21) was analyzed similarly, suggesting that a bend of about 40° is introduced into the major complex (equivalent to C-4, Fig 2A) by gp33^103^ binding. (C) To determine if intermolecular DNA bridges are formed by gp33^103^, two different sized fragments of DNA containing the *33–34* intergenic region (366 bp and 256 bp) were PCR amplified and radiolabeled (S1 Table). The leftmost six lanes contain only the 366 bp substrate, the middle six lanes contain only the 256 bp substrate, and the rightmost six lanes contain both substrates. Complexes formed with each substrate are indicated by long and short arrows respectively. The concentration of protein in each series of substrates is 1) none, 2) 0.54μM, 3) 1.6μM, 4) 5.4μM, 5) 16μM, 6) 54 μM. Protein affinities are shown in Table 1.

To further explore the binding interactions between gp33^103^ and this *33*–*34* control region, we constructed two series of substrates in plasmid pBEND2 [20], one containing only O_R-R_ and one containing the full *33*–*34* intergenic region. We then determined the relative mobilities of complexes formed with gp33^103^ as a function of the position of the sites relative to the ends of the DNA molecules; substrates with a protein-induced bend migrate slowest if the bend is in the center of the substrate (Fig 5). We saw no evidence of DNA bending when only O_R-R_ was present (Fig 5A), but a clear indication of a protein-induced DNA bend with the larger substrate (Fig 5B). No intrinsic bending of *33–34* intergenic region was observed, but bending was indicated in at least two of the protein-DNA complexes (Fig 5B). The magnitude of the overall bend is estimated to be about 40°, although we note that this could arise from multiple protein-DNA interactions. This is consistent with introduction of a bend when gp33^103^ is bound as a tetramer to O_R-L_ and O_R-R_, but it is difficult to exclude the possibility of a hairpin-like DNA loop even though the overall bend is less than might be expected. These observations are also consistent with the interpretation that the relative mobilities of the complexes observed with other substrates are influenced by DNA distortions (Fig 4).

### BPs gp33^103^ does not promote intermolecular bridges

An alternative explanation for the observed gp33^103^ complexes is that gp33^103^ tetramers bind simultaneously to two different DNA molecules to promote intermolecular protein-bridges. To test this, we performed DNA binding assays with two different sized DNA fragments, separately and together (Fig 5C). Both DNA fragments give a similar series of complexes, and when mixed together, we observe only a combination of the complexes formed with the individual substrates, and no new complexes with mobilities suggesting that they contain more than one DNA fragment. We conclude that under these conditions although the complexes may have differing numbers of gp33^103^ protomers, they each contain only a single DNA molecule. We cannot exclude the possibility that bridging interactions are observed under other conditions including high substrate concentration.

### Additional gp33^103^ binding sites in the BPs genome

**Fig 6:**
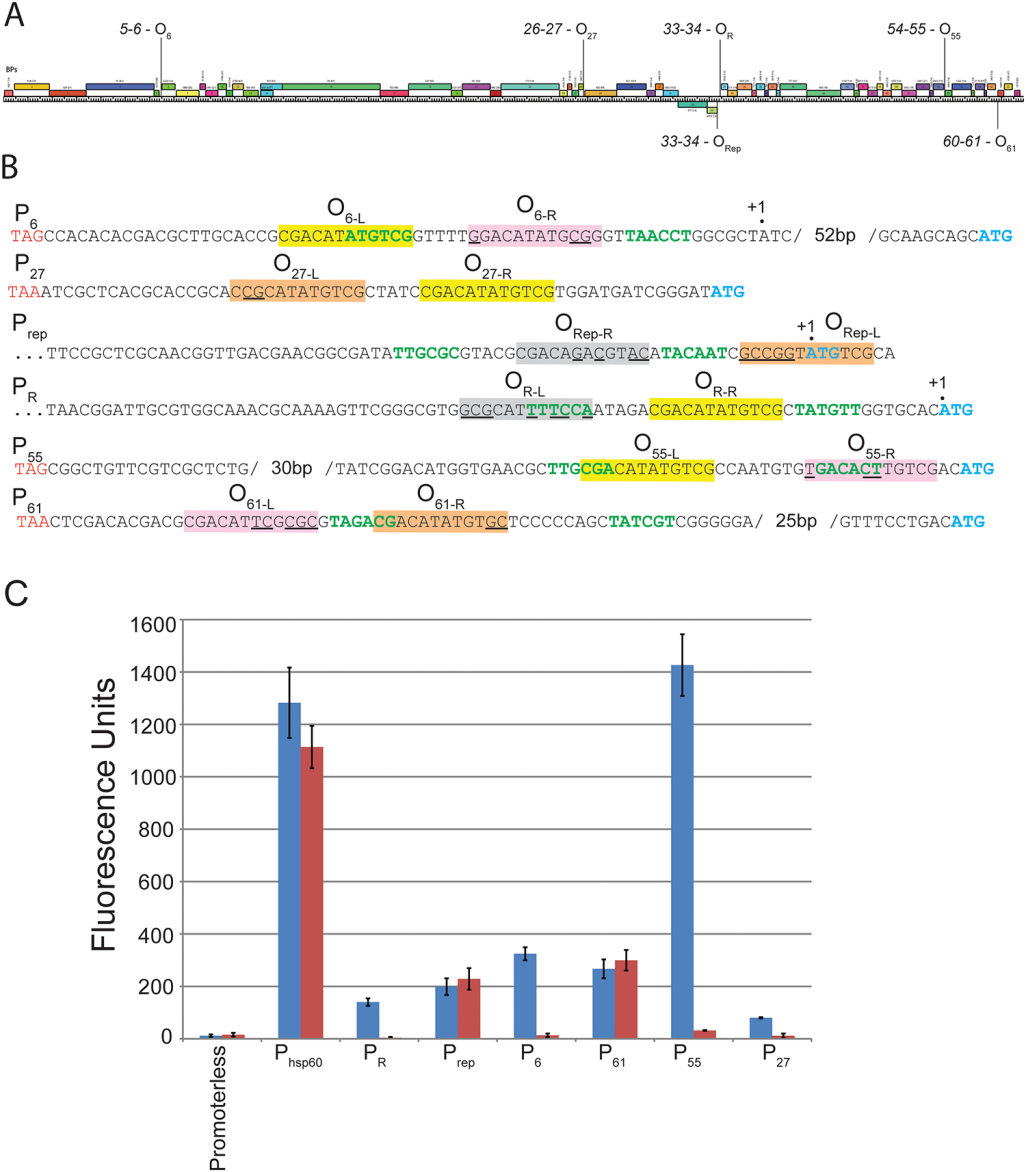
Complete and partial operator sites found throughout the BPs genome. (A) Map of the BPs genome showing open reading frames (colored boxes) with the positions of each of the operator sites indicated. (B) Sequences of the intergenic regions containing operators. Promoter sequences (green text), start codons of open reading frames (blue text), and the stop codons of open reading frames (red text) are shown. Operator half sites corresponding to the 12 bp sequence 5’-CGACATATGTCG are highlighted in yellow. Operator half sites containing sequence departures within these 12 bp sites are highlighted in orange or pink (reflecting gp33^103^ binding or no observed binding respectively; see Fig 7). O_Rep-R_ and O_R-L_ are highlighted in gray. Confirmed transcription start sites are marked as +1 [[13] L. Oldfield and GFH, manuscript in preparation]. (D) Promoter fusions to a mCherry reporter gene were constructed in an integration-proficient vector, transformed into both *M*. *smegmatis* (blue bars) and a BPs lysogen (red bars) and fluorescence levels measured.

BPs gp33^103^ is known to bind to several additional sites within the BPs genome [13] and we investigated whether there are similar complexities to the binding of gp33^103^ at these sites. Three other instances of sequences identical to O_R-R_ are located in small intergenic regions, between genes *5* and *6*, between genes *26* and *27*, and between genes *54* and *55* (Fig 6A and 6B). A site with two base pair differences is located within the *60–61* intergenic region (Fig 6). In view of the proposition that the 12 bp palindrome at O_R-R_ corresponds to a half-site for gp33^103^ binding, we examined the sequences for additional sites related to O_R-R_. Interestingly, in all four regions we identified, different but related sequences are positioned either 5 bp (O_6_, O_27_, O_61_), or 8 bp (O_55_) away from the other half site, consistent with the 12 bp palindrome constituting just one of two half sites. We therefore refer to the left and right halves of each of these as O_6-L_ and O_6-R_, O_27-L_ and O_27-R_, O_55-L_ and O_55-R_, and O_61-L_ and O_61-R_ for O_6_, O_27_, O_55_ and O_61_ respectively (see Fig 6B).

To test whether these regions play a role in the phage transcriptional program and are subject to repressor control, each was inserted upstream of a *mcherry* reporter gene and transformed into *M*. *smegmatis* mc^2^155. The *5*–*6*, *26*–*27*, *54*–*55*, and *60–61* regions all have promoter activity (designated as promoters P_6_, P_27_, P_55_, and P_61_), although the strengths vary considerably, with P_55_ being by far the most active, and P_27_ the weakest (Fig 6C). Putative promoter elements containing -10 and -35 hexameric motifs are predicted for P_6_, P_55_, and P_61_, but not confidently for the weaker P_27_ (Fig 6B). The promoter-reporter plasmids were also transformed into a BPs lysogen and the promoter activities determined (Fig 6C). The P_6_, P_27_, and P_55_ promoters are clearly down regulated in a lysogen, presumably by gp33^103^ [few other phage-encoded proteins are expressed in a lysogen as indicated by RNAseq (LMO and GFH, unpublished observations)] as for the P_R_ control [Fig 6C, [13]]. No regulation for either P_rep_ or P_61_ was observed (Fig 6C).

**Fig 7:**
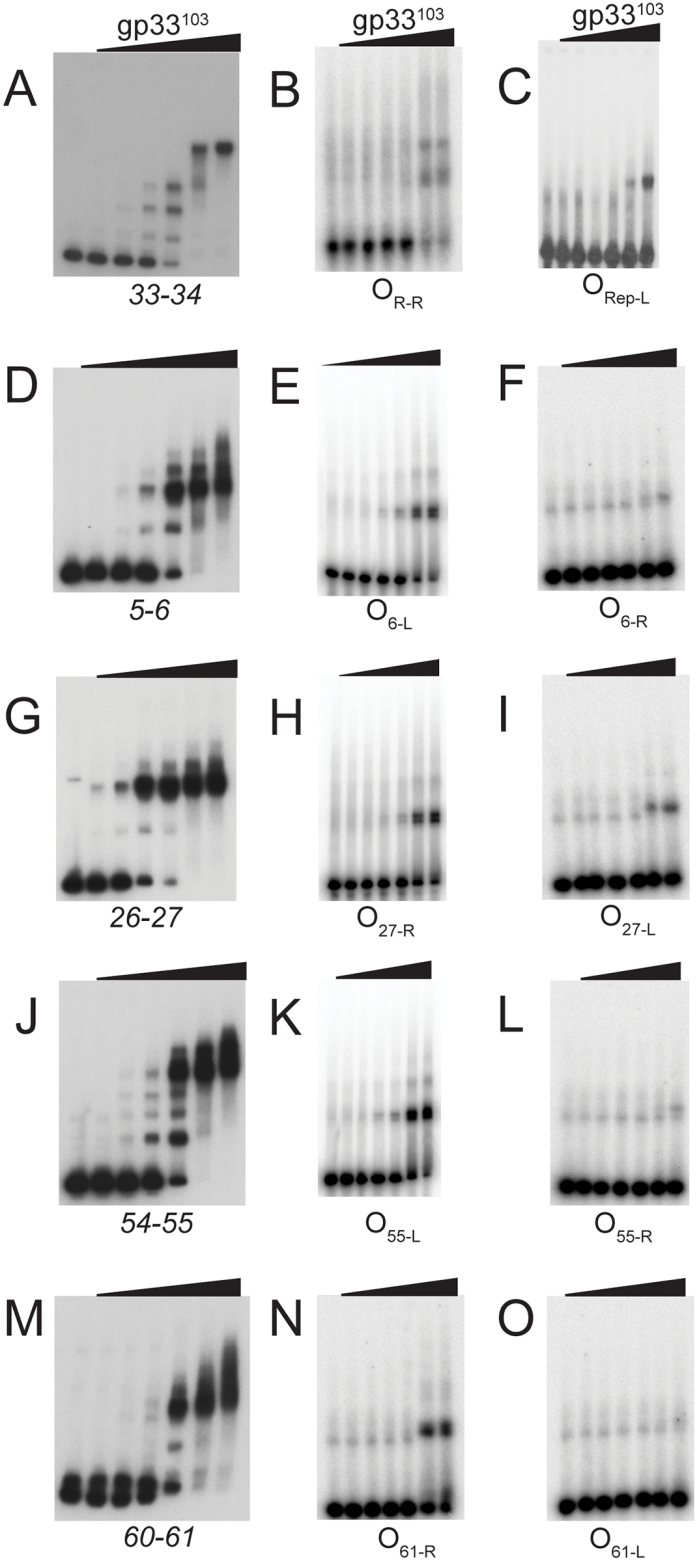
BPs gp33^103^ binding to full and partial operator sites. Binding of gp33^103^ to each of the intergenic regions where full and partial sites are found (see Fig 6; Table 1, S1 Table) are shown in gels A, D, G, J, and M. To examine binding to the O_6-L_, O_27-R_, O_55-L_, and O_61-R_ sites independent from their potential partner sites, 52bp synthetic oligonucleotide substrates were generated with these sites centrally located (S1 Table), and the partner partial sites (O_6-R_, O_27-L_, O_55-R_, O_61-L_) were mutationally ablated to have no sequence similarity to the 12bp palindrome (gels E, H, K and N). Each of the partially conserved partner sites (O_6-R_, O_27-L_, O_55-R_, O_61-L_) were placed in a non-native sequence context (42bp substrates; S1 Table) to test for gp33^103^ binding (F, I, L, and O). Binding profiles of gp33^103^ to O_R-R_ and O_Rep-L_ are shown for comparison (B and C). The concentrations of protein are as follows: 1) none, 2) 0.16μM, 3) 0.54μM, 4) 1.6μM, 5) 5.4μM, 6) 16μM, 7) 54 μM. Protein affinities are shown in Table 1.

Using native gel electrophoresis, gp33^103^ was shown to bind to all four substrates (O_6_, O_27_, O_55_ and O_61_) to generate a single prominent complex and a minor complex with intermediate mobility, with the exception of O_6_ and O_55_ for which additional complexes were observed (Fig 7D, 7G, 7J and 7M). Presumably, the relative simplicity of the binding patterns compared with the *33–34* DNA is because of the lack of additional sites equivalent to O_Rep_. The overall affinities are similar to gp33^103^ binding to the *33*–*34* intergenic region (Fig 7A, Table 1), although binding is about 3-4-fold tighter to O_27_. To dissect out the contribution of the separate potential half sites, substrates were generated in which one half site was specifically ablated so as to leave just the 12 bp palindrome (O_6-L_, O_27-R_, O_55-L_, and for O_61_, O_61-R_, S1 Table), and tested for gp33^103^ binding (Fig 7E, 7H, 7K and 7N). BPs gp33^103^ binds to each of these to form a single complex, but with substantially reduced affinity (Table 1). We then inserted the other half sites (O_6-R_, O_27-L_, O_55-R_, O_61-L_) individually into a common sequence context (S1 Table) and tested gp33^103^ binding (Fig 7F, 7I, 7L and 7O). All of these were bound only very weakly, although complexes were detected with the O_27-L_ substrate (Figs 6B and 7I). These binding data suggest that gp33^103^ binds to DNA containing two half sites, presumably as a tetramer, and the reduced mobility of the complexes formed by gp33^103^ and each full site is slower than for complexes with individual half sites. Although we cannot rule out the possibility that these complexes have different protein-DNA stoichiometries, it is plausible that these differences reflect a protein-induced bend when the full site is bound, as seen at O_R_.

### Binding of gp33^103^ to adjacent 12 bp palindromes

**Fig 8:**
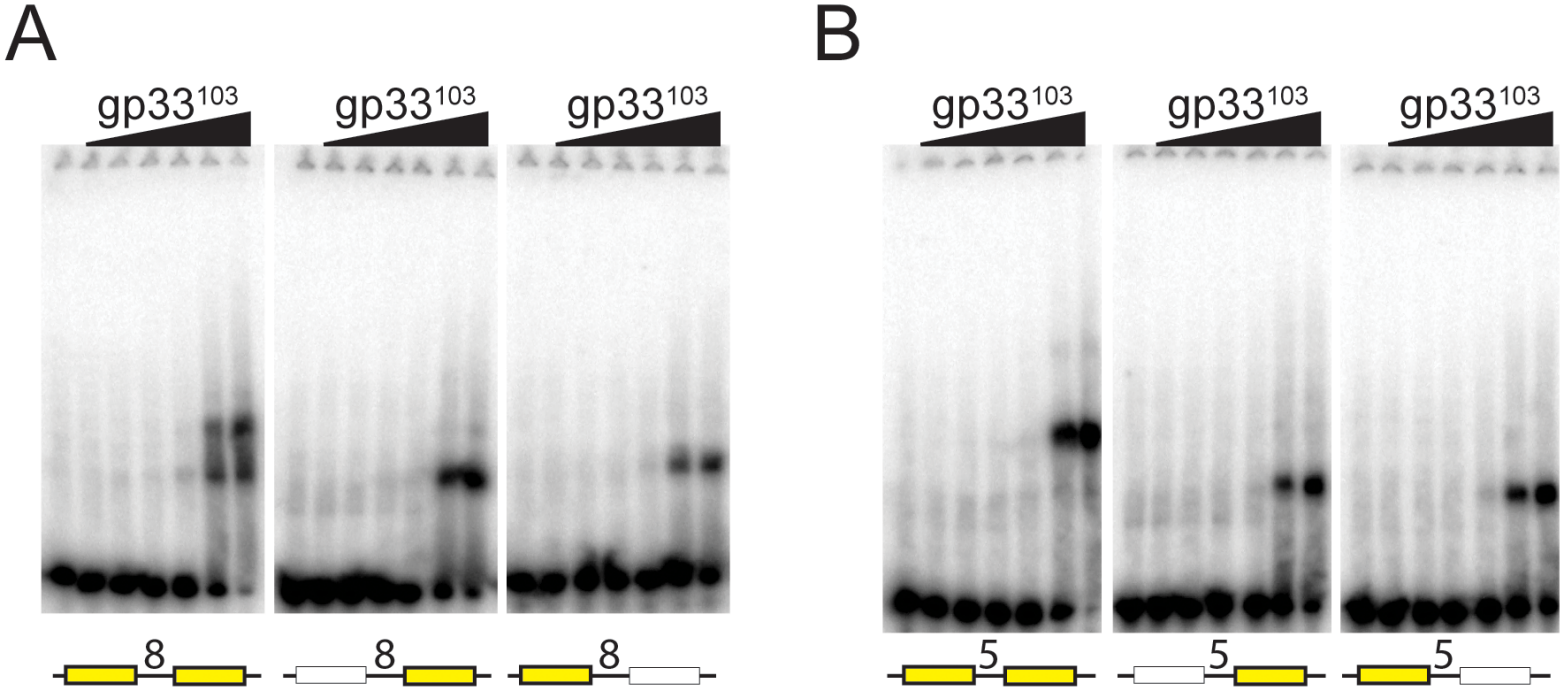
BPs gp33^103^ binds to two adjacent 12 bp palindromes. (A and B) EMSAs showing gp33^103^ binding to substrates containing two 12bp palindromic half sites (yellow boxes) spaced either 8 bp (panel A) or 5 bp apart (panel B). Substrates in which one half site is mutationally ablated is shown as a white box.

To further test the binding of gp33^103^ to two half sites, we generated a series of 42 bp substrates each containing two 12 bp palindromic sequences spaced either 5 bp or 8 bp apart (Fig 8). We also made derivatives of these in which either the left or right half site was mutationally ablated, and examined gp33^103^ binding. With a 5 bp inter-site spacing, BPs gp33^103^ binds to form a single complex which has a slower mobility than those formed when each of the half sites is ablated (Fig 8B). Although the affinities for the three substrates are similar, there is suggestion of cooperative binding to two adjacent sites, as no complex corresponding to single site occupancy is present. A plausible explanation is that the binding energy gained from cooperative binding is balanced by an investment of binding energy into either DNA bending or conformational distortion of the protein. When two 12 bp palindromes are separated by 8 bp (Fig 8A), both the faster and the slower migrating complexes are formed, suggesting lack of cooperative interactions, although the overall affinities are similar to the 5bp spaced substrate suggesting that DNA bending may also be different.

### BPs gp33^103^ binding to mutant DNAs conferring a repressor-insensitive phenotype

**Fig 9:**
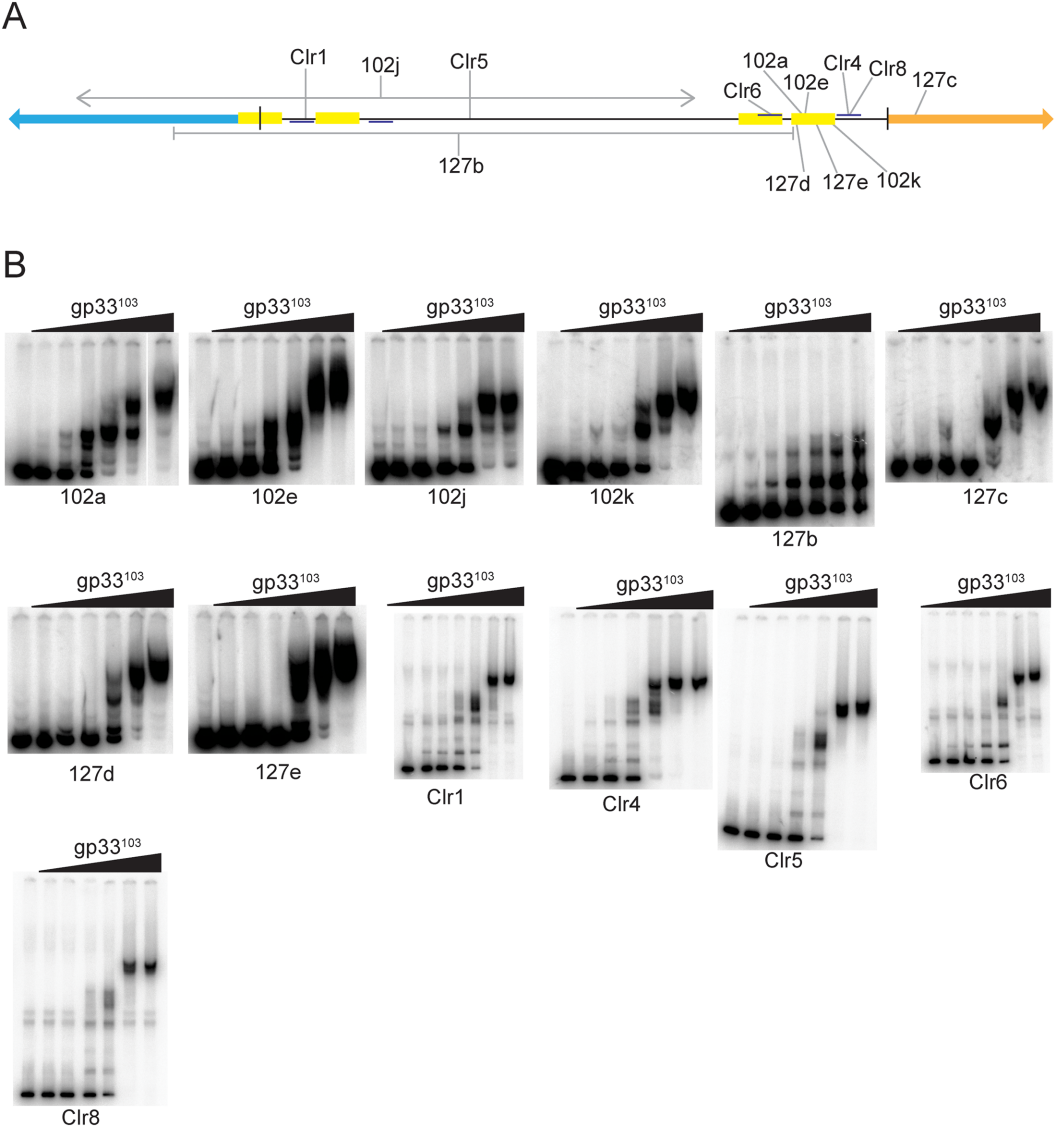
BPs gp33^103^ binding to substrate variants of the *33*–*34* intergenic region derived from repressor-insensitive BPs mutants. (A) Locations of the mutations in each substrate (see Table 1). (B) Binding profiles of gp33^103^ to each *33*–*34* substrate. The concentrations of protein are as follows: 1) none, 2) 0.16μM, 3) 0.54μM, 4) 1.6μM, 5) 5.4μM, 6) 16μM, 7) 54μM. Protein affinities are shown in Table 1.

We previously described the isolation of BPs mutants that are capable of infecting a repressor-expressing strain, and behave as though they are repressor-insensitive. Two such mutants (102a and 102e, Fig 9A) were shown to have point mutations in O_R-R_ that reduce the affinity of gp33^103^ by about 3-10-fold (depending on specific DNA substrate used), apparently sufficient for the commitment to lytic growth to outcompete the resident repressor [13]. Three additional mutants (Clr4, Clr6 and Clr8, Fig 9) contain single substitutions in the regions immediately flanking O_R_ (Fig 9A; S1 Table). Two of these (Clr4, Clr8) are to the right of O_R-R_ in the promoter -10 region such as to influence P_R_ promoter activity, and to give elevated gp34 synthesis which is predicted to promote commitment to lytic growth [13]. One mutant (Clr6) is in the -35 region of P_R_ but is not predicted to influence promoter activity [19]. However, the mutation lies within the putative O_R-L_ site and could therefore influence gp33^103^ binding. We examined gp33^103^ binding to these three mutants (Fig 8) and observed that all three form complexes with similar overall affinity as to the wild-type substrate. One interpretation is that repression of transcription requires a specific tertiary structure, and that point mutations within the binding site influence this structure, although binding affinity may be little different.

We tested the binding of gp33^103^ to other mutants with repressor-insensitive phenotypes, including other point mutations as well as insertions, deletions, and inversions (Fig 9B). Two mutants (Clr1 and Clr5) have mutations distal to O_R_ and we observe that both form complexes with similar relative mobility and affinity as the parent substrate, and the basis for their phenotypes is not clear (Fig 9, Table 1).

Six other repressor-insensitive mutants have more substantial DNA rearrangements in the *33*–*34* region (Fig 9A; Table 1). Four of these (102k, 127c, 127d, 127e) have small duplications near the P_R_/O_R_ region, and gp33^103^ binds to all of them with similar affinity to the wild-type substrate (Fig 9B). However, the profiles of the complexes differ to that of the wild-type, consistent again with the hypothesis that formation of a protein-DNA complex with a specific configuration is important for regulation. A mutant (127b) containing a large deletion and missing much of the intergenic region including O_Rep_ and O_R-L_ but retaining O_R-R_ forms complexes with much faster relative mobility that those seen with other substrates, indicating that perhaps either a monomer or dimer of gp33^103^ is binding, although the basis for such an unusual property is unclear.

## Discussion

We have described here the unusual binding properties of the repressor encoded by mycobacteriophage BPs. The repressor is non-canonical in that it is encoded in two forms, a virally-encoded product 136-residues long (gp33^136^), and prophage-encoded version (gp33^103^) that is 33 residues shorter as a consequence of integrative recombination at the *attP* site situated within the gene *33* open reading frame. The two proteins bind similarly to a DNA substrate containing the regulatory region between genes *33* and *34*, but the binding pattern is complex with multiple protein-DNA complexes observed (Fig 2). The binding affinity is surprisingly weak relative to other phage repressors, although the gp33^103^ and gp33^136^ binding affinities are similar to each other, and other preparations have similar affinities. The gp33 preparations retain five non-native residues at the N-terminus that we have not been able to remove without encountering insolubility, and these could influence the binding affinity. However, we also note that BPs lysogens have high levels of spontaneous lytic induction, which could reflect relatively weak binding of the repressor *in vivo*.

**Fig 10:**
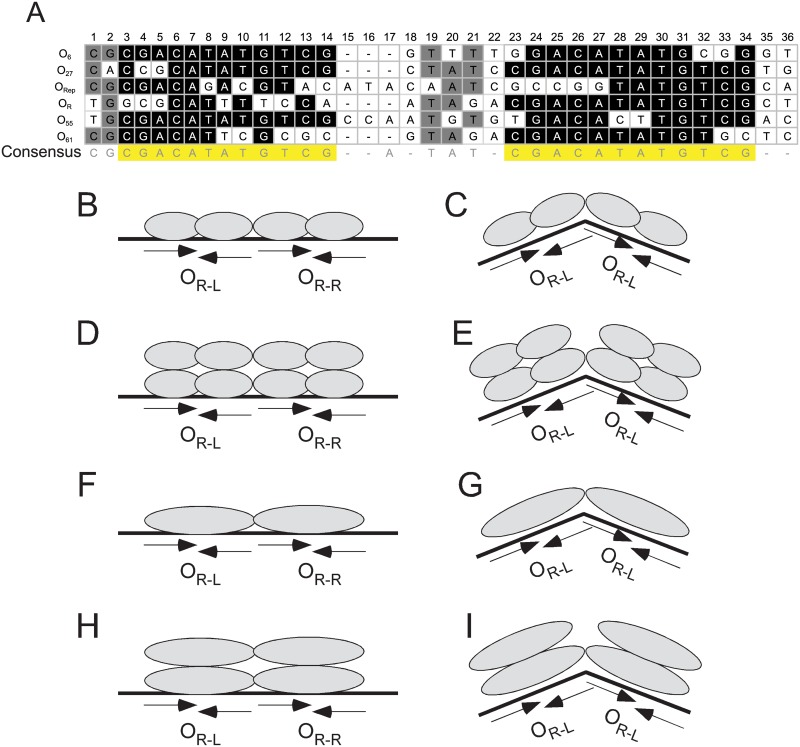
Site alignments and models of BPs gp33^103^ binding. (A) Alignment of all operators found in the BPs genome. Bases in black correspond to the consensus 12bp half site sequences. Bases in gray indicate positions that are present at that position in four or more sites. No shading indicates bases that are present in three or fewer sites. The consensus sequence is shown below the alignment. (B-I) Various plausible configurations of gp33^103^ binding to O_R_ (and related sites) are shown. See text for details.

BPs gp33^103^ binds to a total of five loci within the BPs genome and three promoters (P_6_, P_27_, and P_55_) in addition to P_R_. Alignment of the six putative DNA binding sites (O_6_, O_27_, O_Rep_, O_R_, O_55_, and O_61_) shows the juxtaposition of the half sites within each operator (Fig 10A). In three of the sites (O_6_, O_27_, and O_55_), one half site contains the 12 bp palindrome (O_6-L_, O_27-R_, and O_55-L_) and is easily recognizable, and the other half sites (O_6-R_, O_27-L_, and O_55-R_) contain sequence departures at two or three positions (Fig 10A). At O_61_, both half sites have sequence departures from the consensus (two in O_61-R_ and and four in O_61-L_) although gp33^103^ binds with similar affinity as it does to O_6_ and O_55_ (Fig 7). O_R_ is peculiar in that it contains the easily recognizable O_R-R_, but the other half site is only distantly related, although gp33^103^ clearly binds to it, even in the absence of O_R-R_ (Fig 3). We propose the sequence 5’-GCGCATTTTCCA for O_R-L_ which has only five bases of the 12 bp palindrome (Fig 10A). However, this is spaced five bases away from O_R-R_, which is similar to the geometries of O_6_, O_27_, and O_61_ and which appears to permit cooperative binding (Fig 8). We also note that position eight of this proposed O_R-L_ is part of the P_R_ -35 motif, and substitution with a G base both increases promoter activity, but also reduces the efficiency of repression [19], consistent with the role of O_R-L_ in binding and regulation. The sequences of the O_Rep_ half sites are the most divergent, and the best alignment suggests that O_Rep-L_ and O_Rep-R_ have eight and seven positions conserved respectively, spaced eight bases apart with a geometry similar to that of O_55_. DNase I footprinting is consistent with binding to these sites, and complexes are observed with the small substrate tested that contains the complete sequence (Mt10, Fig 4). At least at higher protein concentrations, binding of gp33^103^ to the *33–34* intergenic regions is expected to involve occupancy of both O_R_ and O_Rep_. Although we cannot exclude the possibility that gp33^103^ forms a DNA loop by simultaneous occupancy of O_R_ and O_Rep_, the 113 bp O_R_-O_Rep_ intersite spacing is substantially below the persistence length of DNA, and DNA looping would likely require either phased A/T tracts to promote DNA bending or an accessory DNA bending protein such as HU.

The nature of the specific protein-DNA interactions suggests there are several plausible models to consider (Fig 10). The previously identified 12 bp palindromic sequence 5’-CGACATATGTCG is typically associated with a related sequence spaced 5–8 bp away, and surprisingly even though the O_R-L_ site is a highly redundant version with only five of the 12 positions conserved (Fig 10A), there is good evidence that gp33^103^ binds to it, even when O_R-R_ is removed (Fig 3C). One set of models includes binding of two protomers to each of the 12 bp sequences within an overall site (e.g. O_R_ or O_27_) (Fig 10B–10E) with the stoichiometry reflecting either a dimer bound to each 12 bp half-site (Fig 10B and 10C), or a tetramer bound to each 12 bp half site (Fig 10D and 10E). The DNA could be relatively straight (Fig 10B and 10D), or could include a modest DNA distortion (Fig 10C and 10E), although we favor the bent DNA models (Fig 10C and 10E) both because of the observed bends seen in Fig 5 and the DNase I enhancement observed at the centers of both O_R-L_ and O_R-R_ (Fig 3A). A second set of models proposes that a single protomer recognizes each 12 bp motif (Fig 10F–10I), with corresponding stoichiometries and bending considerations (Fig 10F–10I) as described for the two-protomer models (Fig 10B–10E). Although 12bp is a somewhat larger segment of DNA than usually recognized by a helix-turn-helix DNA binding motif, it is not unprecedented, and we note that a protomer of γδ resolvase recognizes a similarly-sized half site with contacts spanning both major and a minor grooves of the DNA [21, 22]. The palindromic nature of many of the 12 bp half sites supports two-protomer/half-site models, although we note that the symmetry with the 12 bp sequences is not conserved in all sites.

There is evidence of cooperativity in the occupancy of two 12 bp half sites, but spacing between the sites plays an important role. Cooperative binding appears to be supported by a 5 bp inter-site spacing (Fig 8) as in O_R_, but could occur between single protomers, dimers, or tetramers bound at each half site (Fig 10). However, investment of binding energy into DNA bending and the DNase I hypersensitivity seen within O_R_ and O_Rep_ supports models that include an inter-site bend (Fig 10C, 10E, 10G, and 10I). We note that although models in which a protein dimer (as in Fig 10G) binds to two differently spaced half sites (e.g. 5 and 8 bp) is somewhat unusual, it is observed in the binding of γδ resolvase, where the two half sites within each of three binding sites are separated by 4, 10, and 1 bp respectively [22].

Despite the complexity of the binding profile of gp33^103^ to the *33–34* intergenic region, formation of properly configured complexes is necessary for normal repression in a lysogen. BPs mutants with repressor-insensitive phenotypes that have mutations mapping to the *33–34* intergenic region demonstrate the importance of this sequence and the ability of gp33^103^ to bind to it (Fig 9). Loss of normal repression does not closely correlate with large changes in binding affinity, and it is likely that relatively subtle sequence changes give rise to altered configurations that interfere with repression. This notwithstanding, some DNA substrates of the repressor-insensitive mutants have binding patterns that are more consistent with binding of monomers or dimers (e.g. 127b, Fig 9B), and it is unclear what determines this behavior.

The system of integration-dependent immunity seen in BPs and other phages offers a quite different perspective on phage life style decision making than seen in phage lambda and its relatives, and may represent an ancestral state for temperate phages [12]. It is perhaps not surprising that the repressor has non-canonical binding properties including tetramerization and binding to dispersed sites in the phage genome (Fig 10). Because the repressor can be expressed in two forms that differ in their C-termini, this raises the possibility that the virally-encoded product gp33^136^ forms mixed tetramers with the shorter protein (gp33^103^) and influences functionality even if not binding *per se*. Thus the observed tetramerization and DNA binding profiles may play roles in modulating the overall genetic switch in these phages.

## Materials and Methods

### Expression and Purification of gp33^103^ and gp33^136^

The gp33^103^ and gp33^136^ genes were PCR amplified from a BPs lysate using primers 5’-CAA TCG CCC ATA TGT CGC AAG CAT TCG -3’ / 5’- GAC TAC AAG CTT TCA GAA GGT TGG GGG TTC GA 3’and 5’-CAA TCG CCC ATA TGT CGC AAG CAT TCG -3’/ 5’- TGC CGG AAG AAG CTT TCA CGA CGC TTT ATC C -3’ respectively, which amplified the genes with NdeI recognition sites at the 5’ end of the gene and HindIII recognition sites at the 3’ end. Each gene was cloned into a maltose-binding fusion vector (pLC3) that was linearized with NdeI and HindIII sites for directional cloning, creating two plasmids pVMV20 and pVMV27 for gp33^103^ and gp33^136^ respectively. pVMV20 and pVMV27 were transformed into BL21(DE3)star chemically competent cells (Invitrogen) and grown until cultures reached an OD_600_ of 0.4–0.6. Protein expression was induced with 1 mM IPTG at 17°C overnight. Cells were pelleted and frozen at -80°C. Thawed cell pellets were resuspended in 5mL per gram of Lysis Buffer (50 mM Tris pH 8.0, 500 mM NaCl, 8% glycerol, 1 mM EDTA and 1 mM β-mercaptoethanol) and lysed in 200 mL fractions by sonicating 10 times for 10 sec at 30% output with 30 sec of cooling on ice in between bursts. Pooled cell lysates were cleared by centrifugation at 30,000 x g for 40 min at 4°C. Fusion proteins were extracted from soluble cell lysates using amylose resin affinity chromatography (Invitrogen) and the MBP tag was cleaved from the proteins of interest with TEV protease during overnight dialysis at 4°C. MBP and TEV protease contain C-terminal His tags and were removed from the gp33 proteins using nickel affinity chromatography. The flow through containing pure gp33 proteins was dialyzed into a storage buffer (50 mM Tris pH 8.0, 500 mM NaCl, 50% glycerol, 1 mM EDTA, 1 mM BME) and stored at -20°C.

### DNA Binding Assays

DNA binding assays were carried out according to standard protocols [23]. Briefly, DNA substrates (either PCR substrates or annealed complimentary synthetic oligonucleotides; S1 Table) were 5’ radiolabeled using ATP, [γ-^32^P] with T4 polynucleotide kinase (Roche). Binding reactions contained 5–20 cps radiolabeled DNA probe, 1 μg non-specific calf thymus DNA, and varying concentrations of protein in a binding buffer containing 20 mM Tris pH 7.5, 10 mM EDTA, 25 mM NaCl, 10 mM spermidine, and 1 mM DTT for a total volume of 10 μl. Reactions were incubated at room temperature for 30 min and the resulting protein DNA complexes were resolved on a 5% native gel and detected using autoradiography and a phosphorimaging plate.

### DNase I Footprinting

Footprinting assays were carried out as previously described [23]. Briefly, binding reactions were carried out in a final volume of 50 μl containing various concentrations of protein, 20cps radio labeled probe, 25 mM Tris-HCL pH 8.0, 50 mM KCl, 6.25 mM MgCl_2_, 0.5 mM EDTA, 10% Glycerol, 0.5 mM DTT. Binding reactions were incubated at room temperatures for 30min. After incubation, 50 μl of a solution containing 5 mM CaCl_2_ and 10 mM MgCl_2_ was added to the samples and incubated for 1 minute. Samples were treated with 1.5U DNaseI for exactly one minute, then the digestion reaction was stopped by addition of 90μl of a pre-warmed (37°C) Stop solution (200 mM NaCl, 30 mM EDTA, 1% SDS, 100 μg/mL yeast RNA). Samples were PCI (Invitrogen) extracted and ethanol precipitated. Samples were resuspended in 2–3 μl formamide loading buffer and heated to 95°C for 2 minutes before loading onto a 6% polyacrylamide 7 M urea denaturing gel for resolution. Bands were detected using autoradiography or exposed on a phosphorimageing plate and detected on a FLA-5100; FujiFilm imaging system.

### Size Exclusion Chromatography

Purified gp33^103^ was dialyzed into a buffer containing 10 mM Tris, pH 8.0, 1 mM BME, 500 mM NaCl, and 4.5% glycerol. 1.2 mL of protein was run over a G-120 column using FPLC, 0.5 ml elution fractions were collected and peaks was identified with UV 280 absorbance measurements. Gel filtration molecular weight markers (Sigma-Aldrich) were resuspended in the same buffer at manufacturer recommended concentrations and run over the same column. The molecular mass for the gel filtration standards were plotted against elution volume (Ve) over void volume (Vo) to determine the molecular mass of gp33^103^ based on its elution volume.

### Fluorescent Reporter Assays for Promoter Strength

The vectors were constructed by creating transcriptional fusions of regions of the BPs genome with predicted promoter elements to a codon-optimized mCherry fluorescence gene. The promoter-mCherry vectors were transformed into electrocompetent *M*. *smegmatis* mc^2^155 and a BPs lysogen of *M*. *smegmatis* mc^2^155, as previously described [24]. Fluorescence assays were performed as previously described [19]. Briefly, transformants were grown in biological triplicates under selection shaking at 37°C for 48 hours. From these cultures, 50 μl was aliquoted into 96-well plates (Falcon). Fluorescence was detected at 532 nm (FLA-5100; FujiFilm) and normalized to the optical density at 595 nm (EL800 Universal Microplate Reader; Bio-Tek Instruments) of the aliquot to account for cell density. Fluorescence units were reported as (LAU)/mm^2^)/OD_595nm_. Graphs display the mean fluorescence units ± 95% confidence interval.

## Supporting Information

S1 Fig DNase I footprinting of the *33–34* intergenic region. DNase I footprint used the same substrate and conditions as shown in Fig 3A, except that this is a separate experiment with a clearer DNA ladder.
(PDF)

S1 Table Sequence of DNA substrates used in EMSAs. (DOCX)
