# Supplementary figures and images for: An Unusual Phage Repressor Encoded by Mycobacteriophage BPs
Source: PLoS One. 2015 Sep 2;10(9):e0137187. doi: 10.1371/journal.pone.0137187 (PMC4557955; doi:10.1371/journal.pone.0137187)

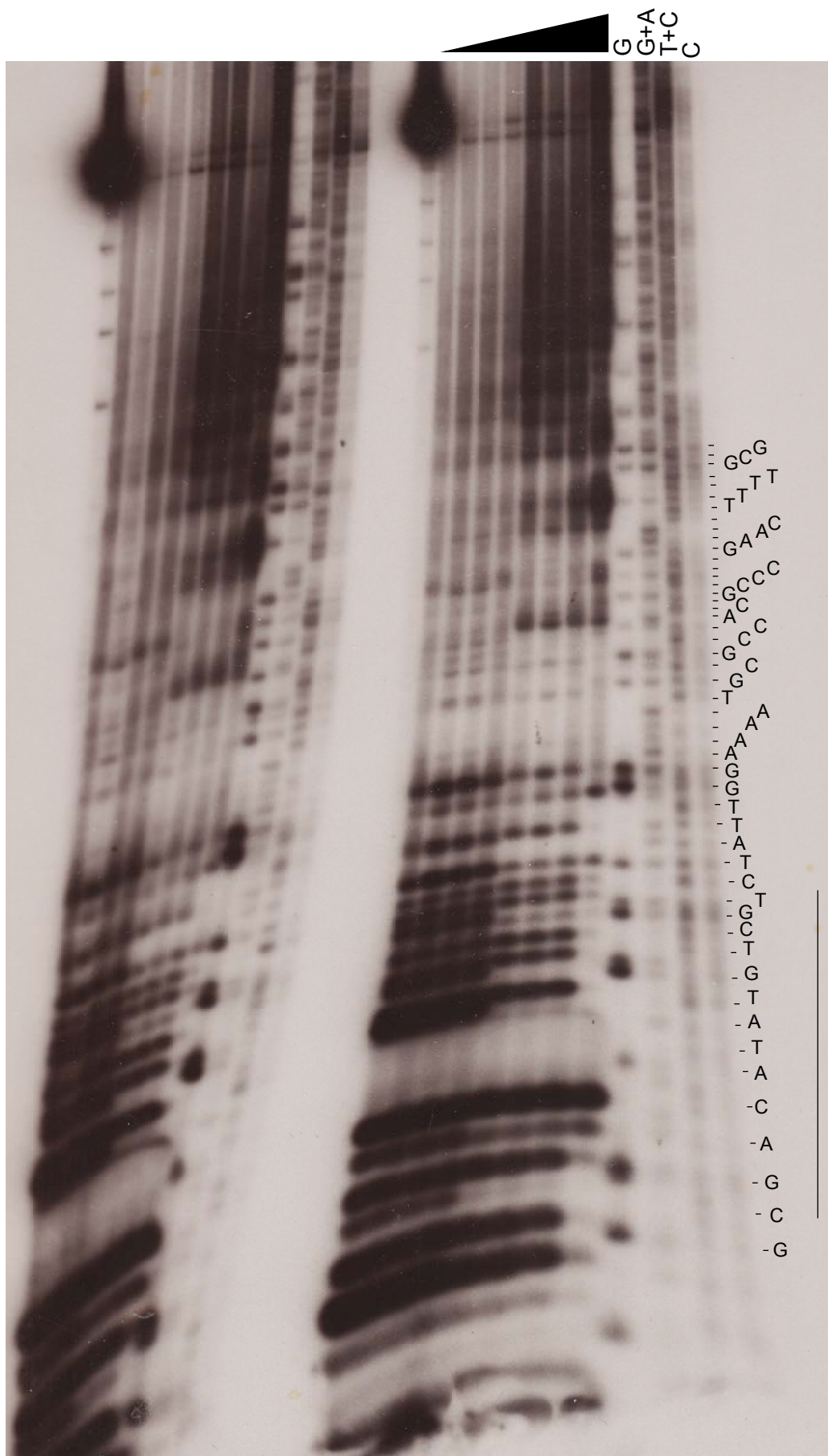

Figure S1

Supplement: S1 Fig — DNase I footprint used the same substrate and conditions as shown in Fig 3A, except that this is a separate experiment with a clearer DNA ladder. (PDF) [file pone.0137187.s001.pdf]
