# Supplementary material for: An Unusual Phage Repressor Encoded by Mycobacteriophage BPs
Source: PLoS One. 2015 Sep 2;10(9):e0137187. doi: 10.1371/journal.pone.0137187 (PMC4557955; doi:10.1371/journal.pone.0137187)
Supplement: S1 Table — (DOCX) [file pone.0137187.s002.docx]

**S1 Table: Sequence of DNA substrates used in EMSAs**

| **DNA Substrate Name** | **Sequence/Primers used** |
| --- | --- |
| *33-34* intergenic region – Fig. 2 | Fwd: GTTCTTCTTGCTTCAAACCAGCTTCAAGG (starts at position 29,228 in BPs genome)  Rev: GTGTAGTGATCGCTTCGGAATGTCATGC (ends at position 29,593 in BPs genome) |
| DNase Footprinting substrate – Fig. 3 | Fwd: TACGAAtctagaACCGGCGATTGTATGTAC  Rev: AGCTCC aagcttGTGCACCAACATAGCGAC |
| Region 1 – Fig. 3 | **AACCGTGCATATCGCGCAATACA**CTTGCGACAT**ACCGGCAACGTTGAAACAATCACGTTG** |
| Region 2 – Fig. 3 | **AACCGTGCATATCGCGCAATACAAAC**ATTGTATG**GTTGAAACAATCACGTTGCACGTTGC** |
| Region 3 – Fig. 3 | **AACCGTGCATATCGCGCAATACAAAC**TTCGGGC**GTTGAAACAATCACGTTGCACGTTGCA** |
| Region 4 – Fig. 3 | **AACCGTGCATATCGCGCAATACAAAC**GGCGCAT**GTTGAAACAATCACGTTGCACGTTGCA** |
| Region 5 – Fig. 3 | **AACCGTGCATATCGCGCAATA**AATAGACGACATATG**TCGCAAACGTTGAAACAATCACGT** |
| TIR-1 – Fig. 3 | ACCGGCGATTGTATGTACGTCTGTCGCGTACGCGCAATAT |
| TIR-2 – Fig. 3 | CGCGCAATATCGCCGTTCGTCAACCGTTGCGAGCGGAAAC |
| TIR-3 – Fig. 3 | GAGCGGAAACGGCACGGCGCGTTGATCCTGGCAAACCGTC |
| TIR-4 – Fig. 3 | GCAAACCGTCGGTGGTAACGGATTGCGTGGCAAACGCAAA |
| TIR-5 – Fig. 3 | CAAACGCAAAAGTTCGGGCGTGGCGCATTTTCCAATAGAC |
| TIR-6 – Fig. 3 | TCCAATAGACGACATATGTCGCT |
| Mt-2 – Fig. 4 | Fwd: TTGGTGCACATGACCCCAACCGAAAC  Rev: GTGTAGTGATCGCTTCGGAATGTCATGC |
| Mt-3 – Fig. 4 | Fwd: TTCGGGCGTGGCGCATTTTCCAATAGA  Rev: GTGTAGTGATCGCTTCGGAATGTCATGC |
| Mt-4 – Fig. 4 | Fwd: TGGCAAACCGTCGGTGGTAACGGAT  Rev: GTGTAGTGATCGCTTCGGAATGTCATGC |
| Mt-5 – Fig. 4 | Fwd: GTTCGTCAACCGTTGCGAGCGGAAA  Rev: GTGTAGTGATCGCTTCGGAATGTCATGC |
| Mt-6 – Fig. 4 | Fwd: ACATACCGGCGATTGTATGTACGTCTGTC  Rev: GTGTAGTGATCGCTTCGGAATGTCATGC |
| Mt-7 – Fig. 4 | Fwd: ACGAAAGCCTGCTCGGCGGGATC  Rev: GTGTAGTGATCGCTTCGGAATGTCATGC |
| Mt-8 – Fig. 4 | Fwd: GTTCTTCTTGCTTCAAACCAGCTTCAAGG  Rev: GTTTCGGTTGGGGTCATGTGCACCAA |
| Mt-9 – Fig. 4 | Fwd: GTTCTTCTTGCTTCAAACCAGCTTCAAGG  Rev: TCTATTGGAAAATGCGCCACGCCCGAA |
| Mt-10 – Fig. 4 | Fwd: GTTCTTCTTGCTTCAAACCAGCTTCAAGG  Rev: ATCCGTTACCACCGACGGTTTGCCA |
| Mt-11 – Fig 4 | Fwd: GTTCTTCTTGCTTCAAACCAGCTTCAAGG  Rev: tttccGCTCGCAACGGTTGACGAAC |
| Mt-12 – Fig. 4 | Fwd: GTTCTTCTTGCTTCAAACCAGCTTCAAGG  Rev: GACAGACGTACATACAATCGCCGGTATGT |
| Mt-13 – Fig. 4 | Fwd: GTTCTTCTTGCTTCAAACCAGCTTCAAGG  Rev: GATCCCGCCGAGCAGGCTTTCGT |
| O_6-L_ – Fig. 7 | CACACACGACGCTTGCACCGCGACATATGTCGGTTTTGCAGACGTGCAGGTT |
| O_6-R_ – Fig. 7 | **GCATTTTCCAATAGA**GGACATATGCGG**CTATGTTGGTGCACA** |
| O_27-R_ – Fig 7 | GCATCACGGACGCTGCTATCCGACATATGTCGTGGATGATCGGGATATGGCC |
| O_27-L_ – Fig. 7 | **GCATTTTCCAATAGA**CCGCATATGTCG**CTATGTTGGTGCACA** |
| O_R-R_ – Fig. 7 | GCATTTTCCAATAGACGACATATGTCGCTATGTTGGTGCACA |
| O_Rep-L_ – Fig 7 | **TTCGGGCGTGGCGCATTTTCCAATAGA**CGACAT**CTATGTTGGTGCACATGACCCCAACCG** |
| O_55-L_ – Fig. 7 | TCGGACATGGTGAACGCTTGCGACATATGTCGCCAATGTGTGCATCTTTGCA |
| O_55-R_ – Fig. 7 | **GCATTTTCCAATAGA**TGACACTTGTCG**CTATGTTGGTGCACA** |
| O_61-R_ – Fig. 7 | **GCATTTTCCAATAGA**CGACATATGTGC**CTATGTTGGTGCACA** |
| O_61-L_ – Fig. 7 | **GCATTTTCCAATAGA**CGACATTCGCGC**CTATGTTGGTGCACA** |
| *5-6* intergenic region – Fig. 7 | Fwd: AACGTAGCCACACACGACGCTTGC  Rev: ATTCGCCATGCTGCTTGCTCCCGGT |
| *26-27* intergenic region – Fig. 7 | Fwd: TGCCGTGGCGCTGATTCTGAGGTAA  Rev: GCATCGGGAAGAAACGATCGGCCAT |
| *33-34* intergenic region (226bp) – Fig. 7 | Fwd: TCCGGCCCATATCGAATGCTTGCGACAT  Rev: CGGTTGGGGTCATGTGCACCAACAT |
| *33-34* intergenic region (366bp) – Fig. 7 | Fwd: GTTCTTCTTGCTTCAAACCAGCTTCAAGG  Rev: GTGTAGTGATCGCTTCGGAATGTCATGC |
| *54-55* intergenic region – Fig. 7 | Fwd: CACGGGGTAGCGGCTGTTCGTCG  Rev: TGCGGGTTCTGGCTGGTGGGGTC |
| *60-61* intergenic region – Fig. 7 | Fwd: GGCGAGTTCGACGCGCCCGT  Rev: TGGCCTTGTTGGTGTCGCGTGGAG |
| Repressor-insensitive BPs mutants -  *33-34* intergenic region – Fig. 9 | Fwd: GTTCTTCTTGCTTCAAACCAGCTTCAAGG (starts at position 29,228 in BPs genome)  Rev: GTGTAGTGATCGCTTCGGAATGTCATGC (ends at position 29,593 in BPs genome) |
| 2 half sites 5bp apart – Fig. 8 | CACCGACGCGACATATGTCGACTGACGACATATGTCGCAGCCTGG |
| Left half site ablated (5bp apart) – Fig. 8 | CACCGACGAAGTGCCACGATACTGACGACATATGTCGCAGCCTGG |
| Right half site ablated (5bp apart) – Fig. 8 | CACCGACGCGACATATGTCGACTGAAAGTGCCACGATCAGCCTGG |
| 2 half sites 8bp apart – Fig. 8 | ACCGACGCGACATATGTCGACGCTTGACGACATATGTCGCAGCCT |
| Left half site ablated (8bp apart) – Fig. 8 | CCGACGAAGTGCCACGATACGCTTGACGACATATGTCGCAGCCTG |
| Right half site ablated (8bp apart) – Fig. 8 | CCGACGCGACATATGTCGACGCTTGAAAGTGCCACGATCAGCCTG |

Sequences corresponding to operator half sites are underlined.

Bold type denotes non-phage sequences
